# Voice Assessment in Patients with Amyotrophic Lateral Sclerosis: An Exploratory Study on Associations with Bulbar and Respiratory Function

**DOI:** 10.3390/brainsci14111082

**Published:** 2024-10-29

**Authors:** Pedro Santos Rocha, Nuno Bento, Hanna Svärd, Diana Monteiro Lopes, Sandra Hespanhol, Duarte Folgado, André Valério Carreiro, Mamede de Carvalho, Bruno Miranda

**Affiliations:** 1 Department of Physiology, Institute of Molecular Medicine, Lisbon School of Medicine, Av. Prof. Egas Moniz, 1649-028 Lisbon, Portugal; 2 Fraunhofer Portugal AICOS, 1649-003 Lisbon, Portugal; 3 Faculty of Medicine and Health Sciences, Linkoping University, 581-83 Linkoping, Sweden; 4 Hospital da Luz—Torres de Lisboa, 1600-209 Lisbon, Portugal; 5 Hospital da Luz Clínica de Odivelas, 2675-671 Lisbon, Portugal; 6 Hospital Beatriz Ângelo, 2674-514 Lisbon, Portugal; 7 LiBPhys (Laboratory for Instrumentation, Biomedical Engineering and Radiation Physics), NOVA School of Science and Technology, 2829-516 Lisbon, Portugal; 8 Department of Neurosciences and Mental Health, Hospital de Santa Maria CHLN, 1649-028 Lisbon, Portugal

**Keywords:** ALS, acoustic analysis, personalized medicine, digital health

## Abstract

Background: Speech production is a possible way to monitor bulbar and respiratory functions in patients with amyotrophic lateral sclerosis (ALS). Moreover, the emergence of smartphone-based data collection offers a promising approach to reduce frequent hospital visits and enhance patient outcomes. Here, we studied the relationship between bulbar and respiratory functions with voice characteristics of ALS patients, alongside a speech therapist’s evaluation, at the convenience of using a simple smartphone. Methods: For voice assessment, we considered a speech therapist’s standardized tool—consensus auditory-perceptual evaluation of voice (CAPE-V); and an acoustic analysis toolbox. The bulbar sub-score of the revised ALS functional rating scale (ALSFRS-R) was used, and pulmonary function measurements included forced vital capacity (FVC%), maximum expiratory pressure (MEP%), and maximum inspiratory pressure (MIP%). Correlation coefficients and both linear and logistic regression models were applied. Results: A total of 27 ALS patients (12 males; 61 years mean age; 28 months median disease duration) were included. Patients with significant bulbar dysfunction revealed greater CAPE-V scores in overall severity, roughness, strain, pitch, and loudness. They also presented slower speaking rates, longer pauses, and higher jitter values in acoustic analysis (all *p* < 0.05). The CAPE-V’s overall severity and sub-scores for pitch and loudness demonstrated significant correlations with MIP% and MEP% (all *p* < 0.05). In contrast, acoustic metrics (speaking rate, absolute energy, shimmer, and harmonic-to-noise ratio) significantly correlated with FVC% (all *p* < 0.05). Conclusions: The results provide supporting evidence for the use of smartphone-based recordings in ALS patients for CAPE-V and acoustic analysis as reliable correlates of bulbar and respiratory function.

## 1. Introduction

Amyotrophic lateral sclerosis (ALS) is a progressive neurodegenerative disease characterized by both upper and lower motor neuron degeneration. This results in progressive muscle atrophy and paralysis, affecting areas such as the bulbar region, impairing speech and swallowing, and the respiratory system (including the diaphragm, thoracic, and abdominal muscles), impairing respiratory function [1,2]. Early bulbar and respiratory dysfunctions are the most devastating variants of the disease associated with shorter survival [3,4,5]. Although most patients initially experience symptoms in their limbs, approximately 85% develop bulbar dysfunction as the disease progresses [6], and usually die from respiratory complications [7,8].

Monitoring disease progression, particularly bulbar and respiratory dysfunctions, remains challenging. The methods currently used in clinical trials and clinical routines mostly rely on subjective rating tools, such as the ALSFRS-R (the revised amyotrophic lateral sclerosis functional rating scale) [9]. Despite the existence of various scales to assess bulbar functional decline [10,11,12], the ALSFRS-R bulbar sub-score remains the only measure routinely employed to evaluate bulbar dysfunction in clinical settings. Moreover, adding to its subjectivity, which limits the sensitivity in tracking the course of the disease, the properties of this individual sub-score have yet to be comprehensively evaluated [13]. On the other hand, volitional lung function tests such as vital capacity (VC), forced vital capacity (FVC), maximal inspiratory/expiratory pressures (MIP/MEP), sniff nasal inspiratory pressure (SNIP), and cough peak flow (CPF) are typically used to assess respiratory function [14,15]. However, while these measures are established and useful for detecting respiratory failure, they require complete patient cooperation and specialized healthcare professionals.

Speech therapists have long utilized acoustic analysis of sound speech features to study bulbar and respiratory dysfunction in various voice disorders [16] primarily because a sound considered “normal” or healthy emerges from a highly coordinated process between the bulbar and respiratory muscles [17]. In fact, in 1968, Darley et al. [18] highlighted the important clinical implications of voice articulation and phonation in assessing neurological diseases, an approach that has been applied to ALS over the past few decades. Research has demonstrated marked differences in specific acoustic parameters in patients with ALS when performing vocal tasks [19,20,21]. Lee et al. [22] identified acoustic patterns for vowels correlating with dysarthria severity. Others have shown differences in features like jitter, shimmer, and harmonics-to-noise ratio (HNR) [20]. The use of speaking rate as an indicator of dysarthria severity and bulbar function deterioration is well recognized [23]. More recently, machine learning analysis using various classification models was employed to evaluate the effectiveness of acoustic parameters in recognizing the presence and severity of ALS [24,25,26]. This type of analysis has also been used to predict FVC values in both patients and healthy controls. However, current studies still rely on specialized equipment or software, making it difficult to adapt for use in home settings [27]. This is particularly crucial for ALS due to the rapid progression of the disease and the numerous hospital visits required by patients.

The emergence of telemonitoring is reshaping the healthcare sector, particularly in enhancing communication between patients, caregivers, and healthcare professionals across a variety of diseases. This technology contributes to diagnosis and facilitates more frequent data collection [28].

Garcia-gancedo et al. [29] successfully showed that a digital platform can remotely gather digital speech characteristics, among other parameters, from patients with ALS, something that was explored using smartphones and mobile applications in research by Rutkove et al. [30] and Connaghan et al. [31]. The results were promising as these approaches, which enable objective data collection at home, were well tolerated. However, it remains to be investigated whether such data correlate with bulbar and respiratory dysfunction, as assessed by clinical tools and evaluations from speech therapists. This research is significant given the convenience of using a simple smartphone.

The main goal of this study was to collect voice recordings from ALS patients, who were asked to read a sentence and sustain a vowel using a smartphone. We analyzed specific sound features extracted from both time and frequency domains and correlated them with the overall functional status and bulbar and respiratory functions. Secondly, we aimed to align this method with a standardized clinical approach by correlating evaluations conducted by a speech therapist with the same clinical variables. The CAPE-V scale (Consensus Auditory-Perceptual Evaluation of Voice), a standardized protocol [16,32] adapted for the Portuguese population [33], was employed as the standard voice assessment tool in this work.

## 2. Materials and Methods

### 2.1. Participants

We included ALS patients observed in our ALS clinic in Lisbon, diagnosed according to the Gold Coast criteria [34]. All patients underwent comprehensive neurological, neurophysiological, neuroimaging, and blood tests to rule out conditions that mimic ALS [35]. Patients with a history of lung disorders, resting dyspnea, laryngeal injury, upper airway infections, significant cognitive involvement impairing their understanding of the phonatory task, or those who declined to participate were excluded. This study was approved by the local research ethics committee of the Centro Académico de Medicina de Lisboa (CAML-Ref. 146/21). All participants provided written informed consent, in accordance with the declaration of Helsinki.

### 2.2. Clinical Evaluation

We collected demographic data, including age, sex, body mass index (BMI), disease duration at the time of study entry, and the region of disease onset. To evaluate the functional disability, we used the ALSFRS-R scale [9]. Bulbar symptoms were quantified using the ALSFRS-R bulbar sub-score, which consists of questions 1 through 3 regarding speech, salivation, and swallowing. Patients with a score less than 12 were considered to have bulbar dysfunction. Sitting predicted forced vital capacity (FVC%) was measured using a computer-based USB spirometer (microQuark®, COSMED®, Rome, Italy), and the best of three reliable maneuvers was used for statistics [4]. In addition to FVC%, predicted maximum expiratory and inspiratory pressures (MEP% and MIP%, respectively) were included as respiratory measures. These tests were performed with the same device (COSMED Pony FX Portable Desktop Spirometer, Rome, Italy) and followed the American Thoracic Society/European Respiratory Society guidelines [36]. Moreover, metrics were calculated using the lung function calculator from ERC.

### 2.3. Voice Sound Recordings and Auditory-Perceptual Assessment

The CAPE-V scale was employed as the voice assessment tool in this study. This scale, validated and adapted to European Portuguese [33], quantifies auditory-perceptual parameters, including severity, roughness, breathiness, strain, pitch, and loudness. Severity represents the overall impression of voice impairment; roughness indicates perceived irregularities in the voice source; breathiness refers to the audible escape of air in the voice; strain is related to the perception of excessive vocal effort; pitch is the perceptual reflection of fundamental frequency; and loudness corresponds to the perceptual reflection of sound intensity [16]. CAPE-V (Supplementary File S1) encompasses three distinct vocal tasks: firstly, participants were instructed to articulate three sustainable vowels (/a/, /i/, and /u/); secondly, they were asked to read six predetermined sentences containing diverse phonetic contexts; lastly, the evaluation involved an assessment of spontaneous speech. To ensure standardization, all subjects were seated in a quiet room and instructed to perform these three specific phonatory tasks. These were recorded according to the prescribed guidelines of CAPE-V. A smartphone (OnePlus, model: BE2013, from OnePlus Technology (Shenzhen) Co., Ltd., Shenzhen, China) was used for the sound recordings; it was positioned at an approximate distance of 20–25 cm from the mouth and an angle of approximately 45°. These measures were implemented to mitigate the influence of wind noise generated when a forceful expulsion of air directly interacts with the microphone [37]. The sound recordings were conducted by one assessor during the patient’s current clinical visit. Each participant underwent a recording session encompassing sixteen distinct sound recordings. Subsequently, four specific recordings—comprising three instances of the vowel /a/ and one spoken sentence—were subjected to objective and comprehensive sound analyses, resulting in a total of 108 recordings analyzed within the context of this study. After data collection, the voice quality assessment was performed by one speech-language therapist, according to the CAPE-V scoring system. Each CAPE-V subcategory was scored using a 100 mm visual analog scale (VAS). The degree of voice quality impairments was evaluated for each vocal variable with a marking along the VAS: the higher the rating, the more severe the impairment (see Supplementary File S2).

### 2.4. Signal Processing and Feature Extraction

Regarding objective analysis, phrase C and vowel /a/ were chosen for a more detailed investigation (Supplementary File S1). This specific phrase was chosen because it includes only voiced phonemes [33]. Vowel /a/ was selected, as it is widely recognized in the literature as suitable for instrumental-based voice features [25,38,39]. For this analysis, the raw signal was first processed with LibROSA—a Python package for audio signal analysis [40]. The analysis was conducted using a frame length of 2048 samples per frame and a hop length of 512. To minimize potential biases stemming from the beginning and end of the recordings, the split function of LibROSA was employed with a cutoff of 20 dB, eliminating the initial and final periods of silence in the voice samples. Once the pre-processing was completed, the generated voice sound signals were analyzed to extract audio-based features. We used the Time Series Feature Extraction Library [41], which extracts over 60 different features on the statistical, temporal, and spectral domains. Considering prior research findings and relevance in general sound analysis, we included the harmonic-to-noise ratio (HNR), jitter (frequency perturbation), shimmer (amplitude perturbation), absolute energy, sound power, entropy, fundamental frequency, spectral bandwidth, speaking rate, and pause time duration. Since three recordings of vowel /a/ were taken, the results considered the mean values of the extracted features. All extracted features were normalized to their maximum value (with a range between −1 and 1).

### 2.5. Statistical Analysis

Data analysis was performed using Python version 3.11.2 (Python Software Foundation, Wilmington, DE, USA). For the significance level, α = 0.05 was considered. Descriptive statistics consisted of frequencies (with proportions) for categorical variables and mean values (with standard deviation) for continuous variables. Parametric tests such as the two-sample *t*-test or one-way ANOVA were applied to compare mean values. If the normality assumption for a continuous variable was violated (indicated by a significant Kolmogorov–Smirnov test with absolute skewness > 2), non-parametric tests such as the Mann–Whitney U-test or Kruskal–Wallis test were considered, and results were reported when they differed from parametric analysis. Linear correlations were used to explore associations between the instrumental-based voice sound features and CAPE-V scores with the ALSFRS-R total score, as well as with pulmonary function measurements such as FVC%, MEP%, and MIP%. Logistic regressions were applied to identify sound features capable of distinguishing between patients with and without bulbar symptoms.

## 3. Results

### 3.1. Demographics and Clinical Characteristics

This study included 27 ALS patients, with 12 presenting bulbar dysfunction. Demographic and clinical variables are shown in Table 1. Age, sex, duration of symptoms, ALSFRS-R, and respiratory variables did not show statistically significant differences (*p* > 0.05) between patients with and without bulbar dysfunction.

### 3.2. Correlations Between Instrumental-Based Voice Features, CAPE-V Scores, and the Disease Functional State

**Figure 1:**
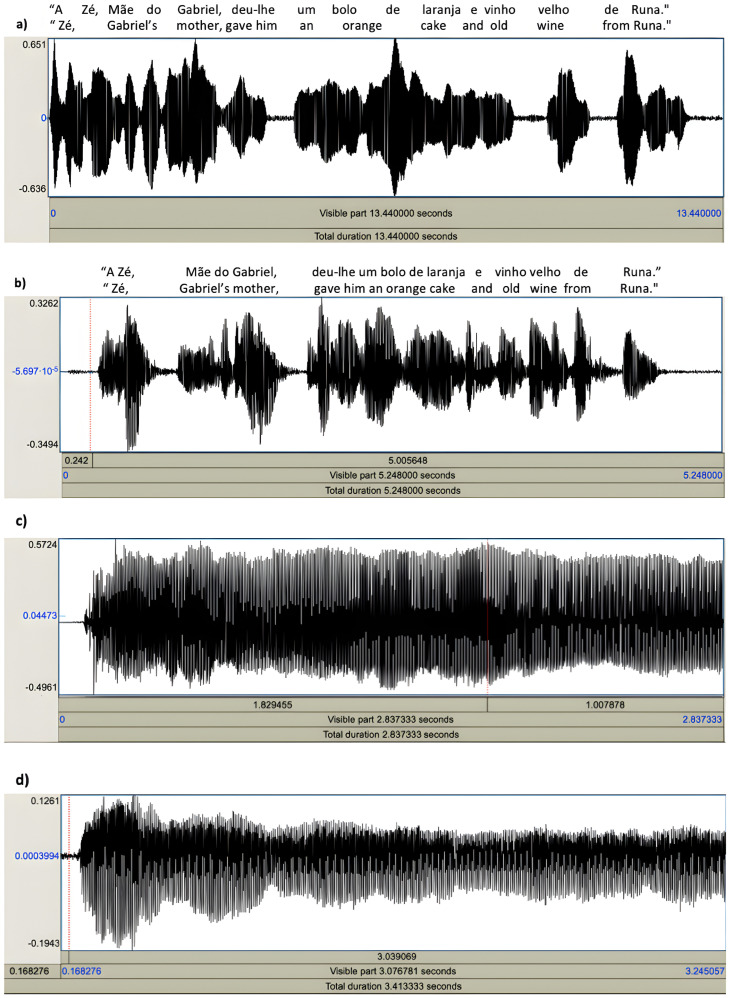
An example of voice sound wave analysis, encompassing the reading of phrase C (**a**,**b**), from the CAPE-V scale, and the sustainable phonation of the vowel /a/ (**c**,**d**); (**a**,**c**) were recorded from a single patient in a more advanced disease state (ALSFRS-R total score of 35), while (**b**,**d**) depict a patient in a less advanced disease state (ALSFRS-R total score of 46). Notably, even though only through visual observation, discernible distinctions between the two tasks emerge, being particularly more evident during the reading of phrase C. The sentence is presented in both Portuguese (the original language) and English to enhance readability.

We investigated the correlation between the ALSFRS-R total score and the voice assessments, including both the instrumental-based voice features and the CAPE-V scores. The lengths of pauses while reading phrase C and its spectral bandwidth demonstrated significant moderate correlations with the ALSFRS-R. Moreover, no significant correlations were found for the CAPE-V sub-scores (see Table 2). Figure 1 presents examples of sound wave patterns generated by two patients in different functional states of the disease.

### 3.3. Correlations Between Instrumental-Based Voice Features, CAPE-V Scores, and the Respiratory Function

Table 3 presents the correlations between the metrics derived from pulmonary function tests and the voice assessments, either by instrumental-based voice collections (extracted from phrase C and vowel /a/) or CAPE-V scores. Regarding the instrumental-based voice assessments, there were significant correlations for FVC%, MIP%, and MEP%. Specifically, for phrase C, the speaking rate and pause time were the features revealing higher coherence with all respiratory measures. We noted that FVC% exhibited a positive correlation with the speaking rate and shimmer while displaying a negative correlation with absolute energy and HNR. MIP% exhibited a positive correlation with the speaking rate and a negative correlation with the length of the pause time. Jitter was also significantly correlated, but it is important to note the difference between this relation and the one with MEP. Lastly, MEP% displayed a significant correlation only with the speaking rate and an inverse correlation with the pause time. Furthermore, for the sustained phonation of vowel /a/, FVC was negatively correlated only with the fundamental frequency and spectral bandwidth. CAPE-V scores did not reveal a significant correlation with FVC%. Nonetheless, the overall severity and the sub-scores for pitch and loudness demonstrated significant correlations with MIP% and MEP% (Table 3). Notably, these correlations were negative—the lower the scores on the CAPE-V assessment, i.e., the lower the pitch and loudness severities—the higher the values of respiratory function variables. This finding indicates an absence of perceived voice quality alterations in patients with better respiratory function.

### 3.4. Voice Sound Features Related to Bulbar Dysfunction

**Figure 2:**
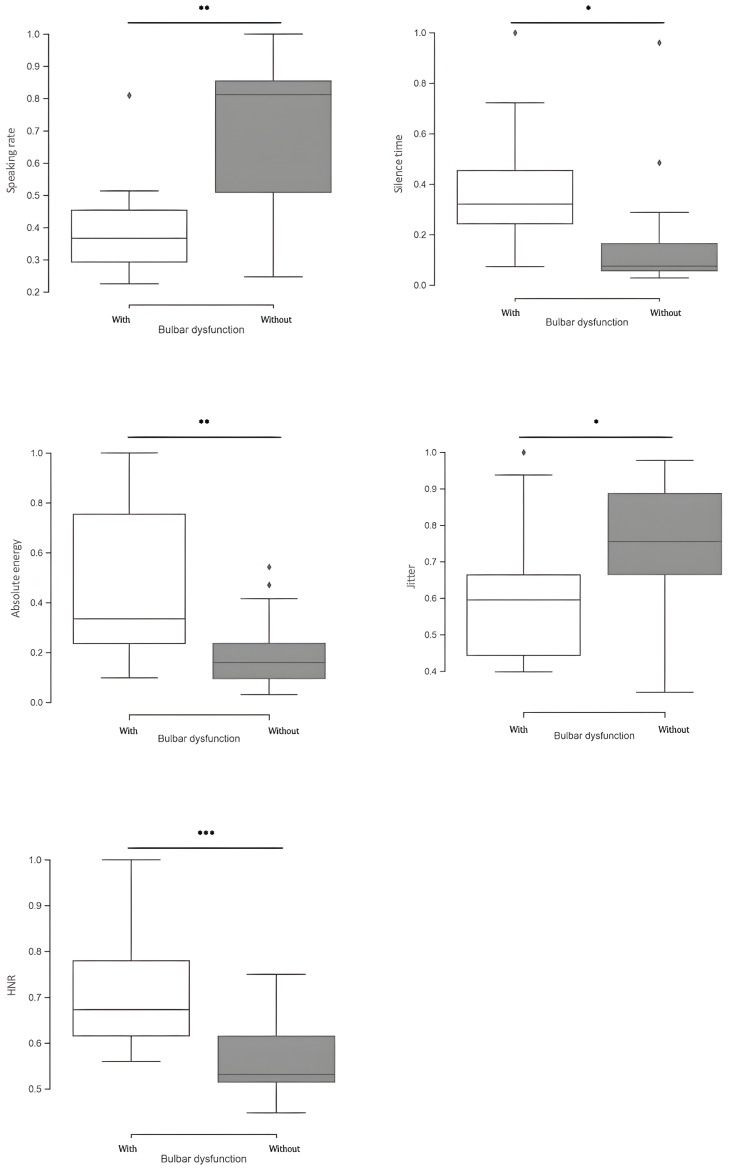
Differences in the normalized instrumental-based voice sound features, extracted from phrase C, between the group patients with (white) vs. without (gray) bulbar dysfunction. In general, patients with bulbar impairments experienced more pronounced effects on their speech, characterized by reduced speaking rates and extended durations of pauses. Correlation is significant at the 0.05 level *, 0.001 level **, and <0.001 level ***.

**Figure 3:**
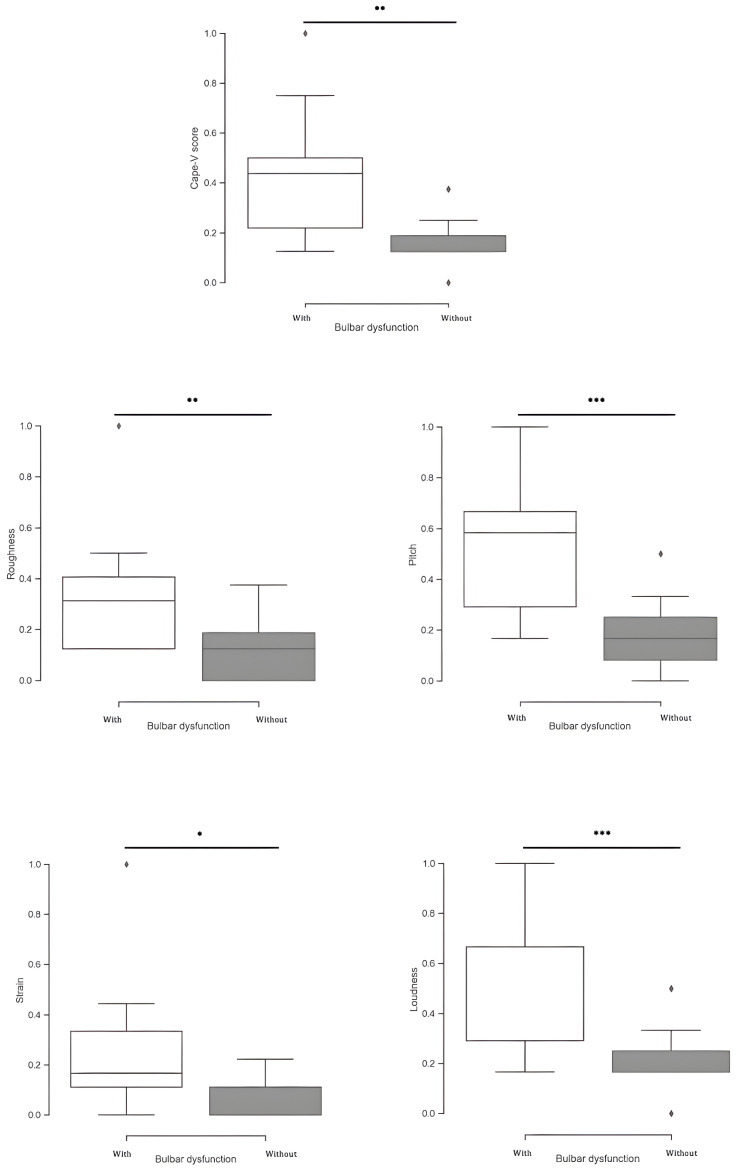
Differences in the normalized CAPE-V scores measures, extracted from phrase c, between the group patients with (white) vs. without (gray) bulbar dysfunction. Correlation is significant at the 0.05 level *, 0.001 level **, and <0.001 level ***.

**Figure 4:**
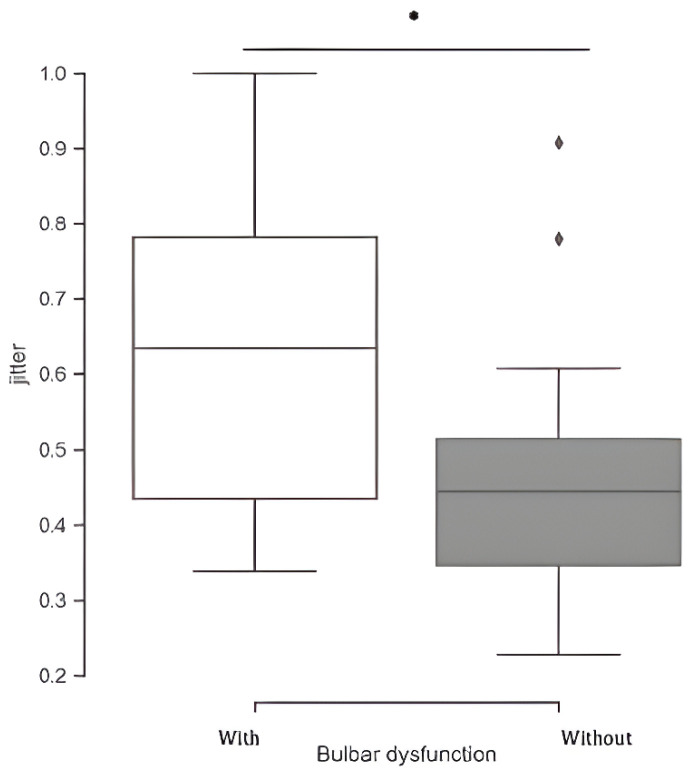
Representation of the normalized jitter, a feature gauging frequency variability, extracted from sustained phonation of vowel /a/, contrasting with the group of ALS patients with bulbar dysfunction (white) vs. without (gray) bulbar dysfunction. Correlation is significant at the 0.05 level *.

We compared ALS patients with and without bulbar dysfunction using instrumental-based voice measurements and the CAPE-V scoring. Regarding the instrumental-based voice features, significant group differences were found for several metrics extracted from phrase C. Patients with bulbar dysfunction showed significantly higher absolute energy (*p* < 0.01) and HNR (*p* < 0.01), while revealing a lower jitter (*p* = 0.043). Moreover, patients with bulbar dysfunction also exhibited a significantly slower speaking rate (*p* < 0.01) and longer pause time (*p* = 0.049) (Figure 2). Applying CAPE-V scores, several differences were disclosed: patients with bulbar dysfunction presented significantly higher scores in overall severity (*p* < 0.01), roughness (*p* < 0.01), strain (*p* = 0.038), pitch (*p* < 0.001), and loudness (*p* < 0.001) (Figure 3). Regarding the sustained phonation of vowel /a/, only the jitter measure was significantly different between the two groups (Figure 4).

### 3.5. Correlations Between the CAPE-V Scores and Instrumental-Based Voice Features

Having identified the associations between instrumental-based voice features and CAPE-V scores with the bulbar and respiratory functions of ALS patients, we examined the correlations between these voice features and CAPE-V scores. The results are presented in Table 4. Interestingly, the CAPE-V scores are only consistently correlated with the speaking rate and pause time of phrase C. Regarding the vowel /a/, significant correlations were observed only in jitter and HNR, specifically with overall severity scores (for both measures), and with pitch and roughness (limited to jitter). Table 5 summarizes the calculated correlations, highlighting those with significant differences.

## 4. Discussion

From a clinical perspective, we found significant correlations between voice sound features and the bulbar and respiratory functions in ALS. Regarding the objective analysis of instrumental-based voice features, particularly those derived from phrase C, we found that they mirrored global functional status (Table 2). In more detail, correlations between speaking rate, pause duration, and the functional state of the disease align with findings from previous studies [24,42,43]. Patients in advanced stages of the disease (with lower ALSFRS-R total scores) exhibit reduced speaking rates and increased pause times. Additionally, this work revealed a positive correlation between the ALSFRS-R total score and the spectral bandwidth (Table 2). In normal, healthy speech, sounds are composed of a combination of different frequencies, and the spectral bandwidth provides insight into the distribution of these frequencies. This finding implies that individuals in poorer functional states often exhibit a more restricted frequency range in their speech compared to those in better states. On the other hand, CAPE-V scores applied by the speech therapist did not correlate with the total ALSFRS-R score (Table 2). Nevertheless, they proved effective in evaluating both bulbar and respiratory impairments, as nearly all the sub-scores enabled the differentiation of patients with and without bulbar impairments, and the CAPE-V’s overall severity, pitch, and loudness were significantly correlated with MIP% and MEP% (Table 3). These positive outcomes were anticipated because voice sound production results from a highly coordinated process between the bulbar and respiratory muscles. Therefore, voice assessment should be sensitive to detect bulbar and respiratory impairments. Similarly, in the analysis of phrase C, instrumental-based voice sound features showed significant correlations with both bulbar symptomatology and respiratory function variables (FVC%, MIP%, and MEP%) (Table 3), particularly the speaking rate, pause time, sound energy, and variables assessing sound variability, such as jitter, shimmer, and HNR. These findings suggest that perceivable loudness and pitch are primarily influenced by the strength of the respiratory muscles, as directly assessed by MIP and MEP), and the measurable characteristics of speech and phonation seem to be predominantly influenced by the exhaled volume and airflow, as evaluated by FVC). These findings offer significant insights into the pathophysiology of respiratory impairments in ALS patients—a topic that deserves further investigation in the future. Jitter, shimmer, and HNR are becoming very prominent, as they have been found sufficient to accurately detect laryngological pathologies using machine learning algorithms [44], as well as bulbar involvement in ALS patients [25,26]. However, they were rarely used to assess respiratory function. Jitter is a measure of frequency perturbation, shimmer is a measure of amplitude perturbation, and HNR represents the ratio between the periodic (vibrations of the vocal cord) and non-periodic elements (glottal noise).

Our work confirms that patients with bulbar impairments have a reduced capacity for varying voice frequency, as shown by lower jitter values (due to the immobilization of bulbar muscles responsible for adjusting and stretching the vocal cords). Moreover, patients with respiratory impairments (indicated by lower FVC) have a diminished capacity for varying sound intensity, as shown by lower shimmer values. Regarding jitter, we found that it was higher in patients with bulbar symptoms during the phonation of the vowel /a/, highlighting the importance of the inherent nature of the task. This finding aligns with the study by Xie et al. (2014) [45], which demonstrated a similar result. We speculate that, when sustained phonation is required, patients with bulbar dysfunction encounter greater challenges in controlling slight variances in sound frequency, especially due to varying properties of the medium (the vocal tract) through which the sound wave travels.

From a technical perspective, we intended to demonstrate the association between frequency-related voice sound features with bulbar dysfunction, and intensity-related voice sound features with respiratory impairment (Table 5, in general). As briefly explained, frequency is perceived by the voice pitch and intensity by its volume. Frequency is very dependent on vocal cord functionality, as it results from its variations, and intensity is very dependent on air volume, which results from respiratory muscle function. Overall, in both subjective and objective evaluations, we found this consistent pattern: jitter, roughness, pitch, and strain values exhibited stronger correlations with bulbar symptomatology, while shimmer, loudness, and pitch showed stronger associations with respiratory impairment. This implies that outcomes such as loss of harmonic complexity narrowed frequency range, and increased regularity or voice sound periodicity are linked with tension or stiffness in vocal cords, significantly restricting the vibrational patterns of sound, and the loss of varying sound intensity linked to abnormal lung function. Absolute energy and HNR demonstrated correlations with both bulbar and respiratory function, providing insights into sound frequency and intensity. It is important to note that while the results were not adjusted for gender, analyses were conducted to determine if there were gender-specific differences in the variables used. Only shimmer, which was higher in men, and HNR, which was higher in women, showed such differences. CAPE-V scores and the remaining instrumental-based voice measures did not exhibit any differences.

Another critical finding involved examining the specific sound wave features on which the subjective evaluation relied on (Table 4). While this type of evaluation is non-invasive, well-tolerated by the patients, brief, and cost-effective, it remains a challenging endeavor due to its subjective nature, as it is influenced by the internal standards of listeners, their background experience, and training. In this work, we found that subjective evaluations, across all sub-scores, heavily depended on intelligibility factors, particularly the speaking rate and pause time, as we found moderate to strong correlations between these metrics and the CAPE-V’s overall severity, roughness, strain, pitch, and loudness. The findings mentioned above reinforce three key points: (1) The extent to which a speaker is comprehensible to a listener is critically important, with speaking rate and pause time being crucial contributors—this is evident even when considering correlations between sound entropy in phrase C, and jitter and HNR in vowel /a/ with CAPE-V’s overall severity; (2) the challenge of accurately evaluating features like fundamental frequency, sound energy, power, and others solely through auditory perception, as shown by correlations between jitter in vowel /a/ and perceptions of roughness and pitch; and (3) the importance of assessing a phrase in combination with a sustained vowel to provide a comprehensive analysis. Thus, subjective analysis should be complemented with a more objective and personalized acoustic analysis, directly related to muscle functionality, which presents an opportunity for future exploration.

Lastly, in the realm of acoustic analysis as a method for detecting voice impairments, there is not only a lack of standardized methodologies, protocols for collecting voice samples or approaches and algorithms for extracting sound features, but, frequently, conclusions are drawn from diverse populations. From a physiological perspective, especially in the context of voice assessment, it is crucial to consider that not all languages share the same phonemes, and even within a single language, phonemes can vary, influenced by factors such as regional differences. Furthermore, reproducing phonemes not present in one’s native language poses challenges, as it requires unfamiliar positioning of the organs responsible for producing speech, such as the lips, oral cavity, tongue, teeth, palate, pharynx, and nasal cavity. This can result in different instrumental-based voice features. This work highlights acoustic analysis in a Portuguese population, which speaks a Latin-derived language.

Finally, smartphones have become important tools for gathering medical and other health-related data to support clinical decision-making. This is particularly relevant in the context of ALS, not only because of the disease’s rapid progression but also due to the physical and psychological burden associated with frequent clinical visits. Rutkove et al. [30] introduced a system incorporating a mobile application designed to collect different data (including speech patterns), which enabled patients to conduct regular self-assessments at home. The study showed improved statistical power on collected data, highlighting the potential of remote monitoring tools. Specifically, the use of smartphones in monitoring voice function holds considerable promise for distinguishing between different clinical manifestations, such as bulbar and respiratory dysfunctions. This capability is beneficial as it may allow clinicians to implement timely and coordinated interventions (as early detection and management may enhance the quality of life and lifespan of ALS patients). Furthermore, this approach could be implemented in centers without access to speech therapists, which would also be beneficial in exploring whether it could serve as a reference policy on initial screenings using smartphones, conducted by clinicians during their routine practice.

### Limitations

The most impactful constraint was the limited sample size, which posed challenges in assessing the generalization of the findings. Specifically, it hindered the possibility of establishing correlations while controlling for various confounding factors, including age, gender, and symptom duration. Moreover, the ALSFRS-R score, especially the definition applied for bulbar dysfunction, is subject to certain limitations, such as being influenced by the subjective perception of symptoms. Therefore, it would be advantageous to compare the analytical methods used between healthy subjects and patients without bulbar impairments. Such a comparison could reveal whether the approach is sensitive to subtle differences that the score may not detect. Considering that the evaluation hinges on perceptual assessment and the experience of the speech-language therapist, it would have also been beneficial to have voice recordings evaluated by multiple specialists to minimize potential errors. Furthermore, the objective analyses of only the phrase C and vowel /a/ can also limit the relationship between this approach and the subjective assessment. Owing to the cross-sectional nature of the study, causal relationships could not be determined. Additionally, exploring correlations between phrenic nerve motor amplitudes and cervical muscle strength presents an interesting, although demanding, scientific opportunity. On the other hand, from a clinical perspective, a potential direction for future research could be to assess whether CAPE-V and acoustic test battery can reliably distinguish whether dysarthria and dysphonia are primarily bulbar, pseudo-bulbar, or mixed; and also, to explore its use on other neuromuscular disorders.

## 5. Conclusions

The present work demonstrates that using a smartphone to collect voice sounds is a useful method for assessing respiratory and bulbar impairments in ALS patients. We find that this approach is well-received by patients, and very convenient, which does not require specialized equipment or handling. This allows researchers to start collecting data from patients’ homes, decreasing the burden of hospital visits, and improving outcomes. This research contributes to the literature by highlighting key sound features that should be prioritized, some of which are quite perceptible to the human ear. However, it is important to note that these analyses should not be the only indicators utilized to evaluate respiratory and bulbar health, as ALS is a multifaceted and intricate disease. Rather, they can be used as adjunct measures, supplementing commonly used tools that monitor disease progression.

## Figures and Tables

**Table 1:** Clinical characteristics of the ALS population.

Clinical Characteristic	ALS Patients (N = 27)
Age (mean ± SD)	60.8 ± 12.6
Gender	
Men	12 (44%)
Women	15 (56%)
BMI (kg/m^2^) (mean ± SD)	23.4 ± 8.2
Symptom duration (months)	
Median	28
1st–3rd Interquartile range	8–141
Disease onset	
Bulbar onset	7 (26%)
Upper limb onset	7 (26%)
Lower limb onset	13 (48%)
ALSFRS-R total score (0–48) (mean ± SD)	39.4 ± 3.1
Bulbar dysfunction	12 (44%)
FVC (%) (mean ± SD)	72.5 ± 16.13

**Table 2:** Pearson (R) and spearman (r) correlation analyses between the ALSFRS-R total score, instrumental-based voice features (extracted from phrase C and vowel /a/), and the CAPE-V scores. Correlation is significant at the 0.05 level *. These values are highlighted in bold.

Metric	R/r Value	*p* Value
**Phrase C**		
Speaking rate	R = 0.37	0.055
**Pause time**	r = −0.40 *	**0.032**
Absolute energy	R = −0.25	0.20
Fundamental frequency	r = −0.31	0.11
Entropy of the signal	R = 0.27	0.18
Power of the signal	r = 0.073	0.71
**Spectral bandwidth**	r = 0.44 *	**0.02**
Shimmer	R = −0.063	0.76
Jitter	R = 0.071	0.69
HNR	R = −0.34	0.082
**Vowel A**		
Absolute energy	R = −0.23	0.24
Fundamental frequency	R = −0.33	0.09
Entropy of the signal	r = −0.26	0.19
Power of the signal	R = 0.23	0.25
Spectral bandwidth	r = 0.22	0.22
Shimmer	r = 0.18	0.36
Jitter	r = −0.13	0.52
HNR	R = −0.19	0.33
**CAPE-V Scores**		
Overall severity	r = −0.34	0.073
Roughness	r = −0.25	0.21
Breathiness	r = −0.26	0.18
Strain	r = −0.18	0.36
Pitch	r = −0.29	0.14
Loudness	r = −0.34	0.064

**Table 3:** Pearson (R) and spearman (r) correlation analyses between pulmonary function tests, the instrumental-based voice features (extracted from phrase C and vowel /a/), and CAPE-V scores. Correlation is significant at the 0.05 level *, 0.001 level **, and <0.001 level ***. These values are highlighted in bold.

	FVC%		MIP%		MEP%	
Voice Features	R/r Value	*p* Value	R/r Value	*p* Value	R/r Value	*p* Value
**Phrase C**						
**Speaking rate**	R = 0.43 *	**0.025**	R = 0.56 **	**<0.01**	R = 0.52 **	**<0.01**
Pause time	r = −0.28	0.15	r = −0.51 **	**<0.01**	r = −0.53 **	**<0.01**
**Absolute energy**	R = −0.51 **	**<0.01**	R = −0.20	0.32	R = −0.058	0.77
Fundamental frequency	r = −0.32	0.10	r = 0.21	0.29	r = 0.049	0.80
Entropy of the signal	R = −0.084	0.67	R = 0.35	0.071	R = 0.38	0.051
Power of the signal	r = 0.32	0.11	r = 0.25	0.22	r = 0.20	0.31
Spectral bandwidth	r = −0.19	0.35	r = 0.19	0.34	r = 0.089	0.65
**Shimmer**	R = 0.48 *	**0.011**	R = 0.24	0.23	R = 0.20	0.31
**Jitter**	R = 0.23	0.22	R = 0.42 *	**0.027**	R = 0.28	0.15
**Harmonic-to-noise ratio**	R = −0.59 **	**<0.01**	R = −0.34	0.086	R = −0.35	0.076
**Vowel A**						
Absolute energy	R = −0.19	0.35	R = −0.10	0.61	R = 0.19	0.35
**Fundamental frequency**	R = −0.54 **	**<0.01**	R = −0.14	0.48	R = −0.21	0.30
Entropy of the signal	r = −0.25	0.19	r = −0.26	0.19	r = < 0.001	0.98
Power of the signal	R = 0.37	0.059	R = 0.25	0.20	R = 0.38	0.052
**Spectral bandwidth**	r = −0.60 ***	**<0.001**	r = −0.19	0.34	r = −0.37	0.058
Shimmer	r = 0.19	0.35	r = 0.17	0.40	r = −0.073	0.71
Jitter	r = −0.22	0.26	r = −0.081	0.68	r = −0.29	0.14
Harmonic-to-noise ratio	R = −0.044	0.82	R = 0.064	0.74	R = −0.28	0.15
**CAPE−V Score**						
Overall severity	r = −0.33	0.097	r = −0.49 *	**0.010**	r = −0.44 *	**0.021**
Roughness	r = −0.30	0.13	r = −0.36	0.062	r = −0.36	0.062
Breathiness	r = −0.36	0.066	r = −0.33	0.085	r = −0.36	0.068
Strain	r = −0.36	0.063	r = −0.12	0.54	r = −0.24	0.22
**Pitch**	**r = −0.33**	**0.093**	r = −0.39 *	**0.042**	r = −0.39 *	**0.044**
**Loudness**	R = −0.38	0.052	r = −0.51 **	**<0.01**	r = −0.48 *	**0.012**

**Table 4:** Correlations between CAPE-V scores and instrumental-based voice features (extracted from phrase C and vowel /a/). Correlation is significant at the 0.05 level *, 0.001 level **, and <0.001 level ***. These values are highlighted in bold.

	Overall Severity		Roughness		Breathiness		Strain		Pitch		Loudness	
Voice Features	r Value	*p* Value	r Value	*p* Value	r Value	*p* Value	r Value	*p* Value	r Value	*p* Value	r Value	*p* Value
**Phrase C**												
**Speaking rate**	−0.53 **	**<0.01**	−0.47 *	**0.014**	−0.24	0.22	−0.37	0.057	−0.50 **	**<0.01**	−0.57 **	**<0.01**
**Pause time**	0.62 ***	**<0.001**	0.54 **	**<0.01**	0.30	0.12	0.47 *	**0.014**	0.64 ***	**<0.001**	0.61 ***	**<0.001**
Absolute energy	0.26	0.18	0.30	0.13	0.082	0.68	0.13	0.50	0.30	0.12	0.31	0.11
Fundamental frequency	−0.24	0.23	−0.027	0.89	−0.27	0.17	−0.067	0.74	−0.070	0.72	−0.23	0.24
**Entropy of the signal**	−0.39 *	**0.043**	−0.30	0.13	−0.10	0.60	0.27	0.17	−0.36	0.065	−0.35	0.076
Power of the signal	−0.020	0.92	−7.60 × 10^−3^	0.97	−0.25	0.21	6.40 × 10^−3^	0.97	−0.076	0.70	−0.034	0.86
Spectral bandwidth	−0.19	0.33	−0.18	0.36	5.80 × 10^−3^	0.97	−0.21	0.29	−0.17	0.40	−0.25	0.20
Shimmer	0.042	0.83	0.079	0.69	0.13	0.51	0.13	0.50	0.095	0.63	−0.041	0.84
Jitter	−0.24	0.22	−0.16	0.41	9.60 × 10^−3^	0.96	0.072	0.71	−0.18	0.36	−0.30	0.13
HNR	0.26	0.19	0.23	0.24	0.12	0.56	0.12	0.55	0.28	0.15	0.33	0.089
**Vowel A**												
Absolute energy	0.019	0.92	0.040	0.84	−0.011	0.95	−0.13	0.51	0.068	0.73	−0.037	0.85
Fundamental frequency	−0.031	0.87	0.081	0.68	0.034	0.86	0.054	0.78	0.10	0.61	1.90 × 10^−3^	0.99
Entropy of the signal	0.14	0.47	0.18	0.34	0.18	0.35	−0.017	0.93	0.17	0.39	0.13	0.48
Power of the signal	0.059	0.76	0.067	0.73	0.071	0.72	0.056	0.77	0.047	0.81	0.059	0.76
Spectral bandwidth	0.015	0.93	−0.0047	0.98	−0.016	0.93	0.099	0.62	7.50 × 10^−3^	0.97	0.010	0.95
**Shimmer**	0.28	0.14	0.33	0.089	0.073	0.71	0.31	0.11	0.30	0.12	0.28	0.16
**Jitter**	0.47 *	**0.013**	0.47 *	**0.012**	0.20	0.30	0.36	0.064	0.52 **	**<0.01**	0.40	0.040
**HNR**	−0.39 *	**0.041**	−0.34	0.086	−0.17	0.39	−0.20	0.31	−0.32	0.10	−0.37	0.054

**Table 5:** Summary of all correlations undertaken in this study. The symbol ‘*’ denotes statistical significance (α = 0.05 was considered).

Voice Features	ALSFRS-R	Bulbar	Respiratory	FVC%	MIP%	MEP%
**CAPE-V Scores**						
Overall severity	-	*	-	-	*	*
Roughness	-	*	-	-	-	-
Breathiness	-	-	-	-	-	-
Strain	-	*	-	-	-	-
Pitch	-	*	-	-	*	*
Loudness	-	*	-	-	*	*
**Phrase C**						
Speaking rate	-	*	-	*	*	*
Pause time	*	*	-	-	*	*
Absolute energy	-	*	-	*	-	-
Fundamental frequency	-	-	-	-	-	-
Entropy of the signal	-	-	-	-	-	-
Power of the signal	-	-	-	-	-	-
Spectral bandwidth	*	-	-	-	-	
Shimmer	-	-	-	*	-	-
Jitter	-	*	-	-	*	-
Harmonic-to-noise ratio	-	*	-	*	-	-
**Vowel A**						
Absolute energy	-	-	-	-	-	-
Fundamental frequency	-	-	-	*	-	-
Entropy of the signal	-	-	-	-	-	-
Power of the signal	-	-	-	-	-	-
Spectral bandwidth	-	-	-	*	-	-
Shimmer	-	-	-	-	-	-
Jitter	-	*	-	-	-	-
Harmonic-to-noise ratio	-	-	-	-	-	-

## Data Availability

The data presented in this study are available upon request from the corresponding author due to data privacy.

## Supplementary Materials

The following supporting information can be downloaded at https://www.mdpi.com/article/10.3390/brainsci14111082/s1, File S1: CAPE-V task protocol; File S2: CAPE-V classification protocol.

## Abbreviations

The following abbreviations are used in this manuscript:

ALS	amyotrophic lateral sclerosis
ALSFRS-R	revised amyotrophic lateral sclerosis functional rating scale
VC	vital capacity
FVC	forced vital capacity
MIP	maximum inspiratory pressure
MEP	maximum expiratory pressure
SNIP	sniff nasal inspiratory pressure
CPF	cough peak flow
HNR	harmonics-to-noise ratio
CAPE-V	consensus auditory-perceptual evaluation of voice
BMI	body mass index
VAS	visual analog scale
