# Supplementary material for: Voice Assessment in Patients with Amyotrophic Lateral Sclerosis: An Exploratory Study on Associations with Bulbar and Respiratory Function
Source: Brain Sci. 2024 Oct 29;14(11):1082. doi: 10.3390/brainsci14111082 (PMC11591699; doi:10.3390/brainsci14111082)
Supplement: Supplementary file 1 [file brainsci-14-01082-s001.zip › brainsci-3229143 Supplementary_file_S1.pdf]

“Consensus Auditory-Perceptual Evaluation of Voice” 2nd European Portuguese Version (II CAPE-V PE)

Voice sample #: ------------

Date: -------------

The following parameters of voice quality will be rated upon completion of the following tasks:

1. Sustained vowels, /a/ and /i/ for 3-5 seconds duration each;
2. Sentence production:
    a. Num domingo esteve sol e fui com o avô António à esplanada “Évora” comer uma empada.
    b. Segundo Simão, só Samuel sabe.
    c. A Zé, mãe do Gabriel, deu-lhe um bolo de laranja e vinho velho de Runa.
    d. É hora da Urraca ir à caça.
    e. Onde eu brinco há um ninho de andorinhas encostado ao muro.
    f. A Kika tapou a tua capa preta.
3. Spontaneous speech in response to: “Tell me about the place where you grew up”.
