# Supplementary material for: Voice Assessment in Patients with Amyotrophic Lateral Sclerosis: An Exploratory Study on Associations with Bulbar and Respiratory Function
Source: Brain Sci. 2024 Oct 29;14(11):1082. doi: 10.3390/brainsci14111082 (PMC11591699; doi:10.3390/brainsci14111082)
Supplement: Supplementary file 1 [file brainsci-14-01082-s001.zip › brainsci-3229143 Supplementary_file_S2.pdf]

Consensus Auditory-Perceptual Evaluation of Voice (CAPE-V)

Voice Sample #:______________________________

The following parameters of voice quality will be rated upon completion of the following tasks:
1. Sustained vowels, /a/ and /i/ for 3-5 seconds duration each.
2. Sentence production:
   a. The blue spot is on the key again.
   b. How hard did he hit him?
   c. We were away a year ago.
   d. We eat eggs every Easter.
   e. My mama makes lemon muffins.
   f. Peter will keep at the peak.
3. Spontaneous speech in response to: "Tell me about your voice problem." or "Tell me how your voice is functioning."

Legend: C = Consistent I = Intermittent
MI = Mildly Deviant
MO =Moderately Deviant
SE = Severely Deviant

							SCORE
Overall Severity	______ MI	MO	SE	C	I	___/100
Roughness	______ MI	MO	SE	C	I	___/100
Breathiness	______ MI	MO	SE	C	I	___/100
Strain	______ MI	MO	SE	C	I	___/100
Pitch (Indicate the nature of the abnormality): ________	______ MI	MO	SE	C	I	___/100
Loudness (Indicate the nature of the abnormality): ________	______ MI	MO	SE	C	I	___/100
__________	______ MI	MO	SE	C	I	___/100
__________	______ MI	MO	SE	C	I	___/100

COMMENTS ABOUT RESONANCE: NORMAL OTHER (Provide description):__________________

____

ADDITIONAL FEATURES (for example, diplophonia, fry, falsetto, asthenia, aphonia, pitch instability, tremor, wet/gurgly, or other relevant terms):

Clinician:____________________
